# A Computational First Principle Examination of the Elastic, Optical, Structural and Electronic Properties of AlRF_3_ (R = N, P) Fluoroperovskites Compounds

**DOI:** 10.3390/molecules28093876

**Published:** 2023-05-04

**Authors:** Amjad Ali Pasha, Hukam Khan, Mohammad Sohail, Nasir Rahman, Rajwali Khan, Asad Ullah, Abid Ali Khan, Aurangzeb Khan, Ryan Casini, Abed Alataway, Ahmed Z. Dewidar, Hosam O. Elansary

**Affiliations:** 1 Aerospace Engineering Department, Faculty of Engineering, King Abdulaziz University, P.O. Box 80204, Jeddah 21589, Saudi Arabia; 2 Department of Physics, University of Lakki Marwat, Lakki Marwat 28420, Khyber Pakhtunkhwa, Pakistan; 3 Department of Mathematical Sciences, University of Lakki Marwat, Lakki Marwat 28420, Khyber Pakhtunkhwa, Pakistan; 4 Department of Chemical Sciences, University of Lakki Marwat, Lakki Marwat 28420, Khyber Pakhtunkhwa, Pakistan; 5 Department of Physics, Abdul Wali Khan University, Mardan 23200, Khyber Pakhtunkhwa, Pakistan; 6 School of Public Health, University of California, 2121 Berkeley Way, Berkeley, CA 94704, USA; 7 Prince Sultan Bin Abdulaziz International Prize for Water Chair, Prince Sultan Institute for Environmental, Water and Desert Research, King Saud University, Riyadh 11451, Saudi Arabia; 8 Department of Agricultural Engineering, College of Food and Agriculture Sciences, King Saud University, Riyadh 11451, Saudi Arabia; 9 Plant Production Department, College of Food and Agriculture Sciences, King Saud University, Riyadh 11451, Saudi Arabia

**Keywords:** condense matter, fluoro-perovskite, visual properties, structural properties, electronic properties

## Abstract

This work describes an ab initio principle computational examination of the optical, structural, elastic, electronic and mechanical characteristics of aluminum-based compounds AlRF_3_ (R = N, P) halide-perovskites. For optimization purposes, we used the Birch–Murnaghan equation of state and discovered that the compounds AlNF_3_ and AlPF_3_ are both structurally stable. The IRelast software was used to compute elastic constants (ECs) of the elastic properties. The aforementioned compounds are stable mechanically. They exhibit strong resistance to plastic strain, possess ductile nature and anisotropic behavior and are scratch-resistant. The modified Becke–Johnson (Tb-mBJ) approximation was adopted to compute various physical properties, revealing that AlNF_3_ and AlPF_3_ are both metals in nature. From the density of states, the support of various electronic states in the band structures are explained. Other various optical characteristics have been calculated from the investigations of the band gap energy of the aforementioned compounds. These compounds absorb a significant amount of energy at high levels. At low energy levels, the compound AlNF_3_ is transparent to incoming photons, whereas the compound AlPF_3_ is somewhat opaque. The examination of the visual details led us to the deduction that the compounds AlNF_3_ and AlPF_3_ may be used in making ultraviolet devices based on high frequency. This computational effort is being made for the first time in order to investigate the aforementioned properties of these chemicals, which have yet to be confirmed experimentally.

## 1. Introduction

As the world’s population is growing day by day, it is causing energy consumption to increase across the world. This results in a decrease in energy. For this purpose, new sources of energy, which are free from environmental problems, are necessary. Solar energy is renewable, does not cause environmental pollution, is available on a large scale and can be used as an excellent replacement for non-renewable energy sources. In recent years, numerous approaches for capturing solar energy have been developed, including solar architecture, solar heating, artificial photosynthesis, photovoltaics and photocatalytic water splitting. Among others, photovoltaics, which harness solar energy and utilize photovoltaic effects to turn sunlight into electricity, have drawn a significant amount of interest. A photovoltaic device known as a solar cell uses the sun’s energy to generate electricity. The solar chambers are typically divided into three age groups. The first age group of solar cells is wafer-based, the second compeers are based on thin film and the third group uses carbon-based assemblies. The first and second group of solar booths have been recycled for a long time, but their use has been constrained by their high cost, difficult manufacturing processes and negative environmental impact. Thus, researchers are searching for novel, inexpensive and pollution-free materials for solar cells. A variety of solar cell types, including multi-crystal silicon (mc-Si cells) and mono-crystal silicon solar chambers (c-Si cells), CIGS solar chambers, CdTe-based solar cells, quantum-dot-sensitized solar cells, organic photovoltaics and perovskite solar chambers, have all been described so far. To be exact, the cost of the materials and the efficiency of power renovation are important factors in the commercialization of solar cells. With considerable power conversion efficiency (PCE) of 25–26%, the third group of silicon-based solar cubicles had dominated up until this point. Perovskite solar chambers, a novel class of third-generation solar booths that demonstrate a PCE of 22.1%, are an alternative to silicon solar cells. Since the discovery of perovskites, they has remained a great source of interest for material scientists. Perovskites exhibit fascinating qualities, which is why they have tremendous use in nanotechnology, particularly in the field of nano-structured solar cells. A class of substances with a peculiar crystal structure, made up of cubic and diamond forms, is referred to as “perovskite”. Its superconducting, electronic and ferroelectric characteristics are very interesting and attractive, catching the attention of researchers.

Research interest in perovskite solar cells has increased dramatically since it was revealed that they are extraordinarily effective at absorbing light that may be turned into electricity. A tremendous amount of work has been conducted in this field for the creation of new perovskite compounds with outstanding characteristics. Perovskites have revealed a number of interesting features, including superconductivity, enormous magnetoresistance, spin-dependent-transport (spintronics) and catalytic abilities, explaining how the particles or combination of particles are active in the assembly. As an outcome, perovskites provide an interesting research environment for scientists. Perovskites are mostly present in nature as oxides formed with silicates (such as bridgmanite rocks). They can also be found in nature as fluoride, chloride, hydroxide, arsenide and intermetallic complexes. Although there are not many naturally occurring perovskite minerals, synthetic perovskites have an elemental makeup that covers the entire periodic table and a wide range of complex formulae, including metallic perovskites, hybrid organic–inorganic perovskites, metal-free perovskites and even noble gas-based perovskites.

Compounds with the chemical formula ABF_3_ have a fluoro-perovskite structure. In such halide perovskite structures, the cation A has 12 halide atoms bound to it, and the other cation, represented by B, has 6 fluorine atoms connected to it. Fluoro-perovskite materials are a distinct class of compounds which have solid crystalline characteristics and exceptional electronic properties, ranging from insulators to semiconductors. In the past few years, these compounds received significant attention due to their numerous applications in the field of radiation dosimeters, material scintillation, the semiconductor industry and as a lens material in photonic lithography [1,2,3]. The results of a large number of studies concluded that these complexes are usually stable mechanically and elastically anisotropic [4,5,6]. Uses of the ABF_3_ composite include high-efficiency photovoltaic and energy storage systems used in the automobile and electronics industries [7,8]. To generate stable fluoro-perovskites, F (fluorine) is normally coupled with other compounds, such as transition metals and organic and or inorganic compounds. Fluoro-perovskites with large band gaps are the best options. These components may be used to produce complicated lattice-matched compounds with large band gaps, permitting lattice-matching and group space engineering [1]. The huge energy band gap is a property shared by several substances. Because of their great potential and low absorption edges, these compounds may be employed as glass in vacuum ultraviolet and ultraviolet wavelengths [9,10,11]. Recently, some research work on such compounds has been published, which may be referred to in the references section [12,13,14]. Harmel et al. [15] utilized DFT to investigate various properties of BaCsF_3_ fluoro-perovskites, finding that CsBaF_3_ may be useful for optoelectronic devices because of its wide direct bandwidth and groups of the unreal component of the shielding properties in the ultraviolet field. Daniel et al. [16] investigated the important properties of LiBaF_3_ and discovered that these chemicals are more suitable for use in preparing devices for storing energy.

The new ternary family compound AlRF_3_ (R = N, P) has the capacity to be used as a visual material in current electronic technologies. The composites that have forbidden a region width wider than 3.10 eV show better results in the ultraviolet spectrum. Because they are electrical conductors with great transparency across a small range of energies, AlNF_3_ and AlPF_3_ compounds exhibit metallic characteristics and are ideal candidates for electrical applications. The basic goal of the current research effort is to practice the DFT and the FP-LAPW approach for investigating the fundamental characteristics, such as the electronic, elastic, optical and structural properties of AlRF_3_ (R = N, P) fluoro-perovskites, which may be used as core data for upcoming experimental research efforts on the materials listed above.

## 2. Computational Methodology

The full potential linearized augmented plane wave (FP-LAPW) technique was recycled to calculate the data for these substances using the coding computer application WIEN2K [17,18,19,20,21,22]. The electronic and other parameters, such as optical, density of state and so on, were calculated using the Tran–Blaha modified Becke–Johnson exchange potential approximation (TB-mBJ) technique. GGA approximation was used to calculate the exchange–correlation potential, but this method resulted in an incorrect band gap value and needed to be adjusted using either the TB-mBJ method or GGA plus the multi-orbital mean-field Hubbard potential (GGA + U). The optical characteristics and band gap calculated using these methods corresponded well with the experimental data [23,24]. In this research, we looked into the particular FP-LAPW technique with the smallest radii in muffin-tin spheres (RMT), which was equal to eight, and, in our case, K_max_, which was the norm of the most extreme K in the plane wave expansion. R = (N, P) elements and F had RMTs of 1.64, 1.77 and 2.06 au. The Fourier extended charge density was reduced to G_max_ = 13.0 au in the muffin-tin region, while the spherical harmonics were extended to l_max_ = 11.0. The self-consistent field values were observed to have been satisfied when the total energy was within the range of 0.001 Ry. Birch–Murnaghan’s equation of state [25] allowed us to draw a graph of energy vs. volume, from which we extracted the structural variables. The elastic constants (ECs) of our chosen crystals were obtained using the computer program IRelast, and the compounds’ elastic properties were then calculated [26]. The dielectric permeability was also recycled to assess the visual characteristics [27,28].

## 3. Results and Discussion

This segment of the study includes a full technical explanation of the results obtained utilizing the Tb-mBJ potential approaches.

### 3.1. Structural Properties

**Figure 1:**
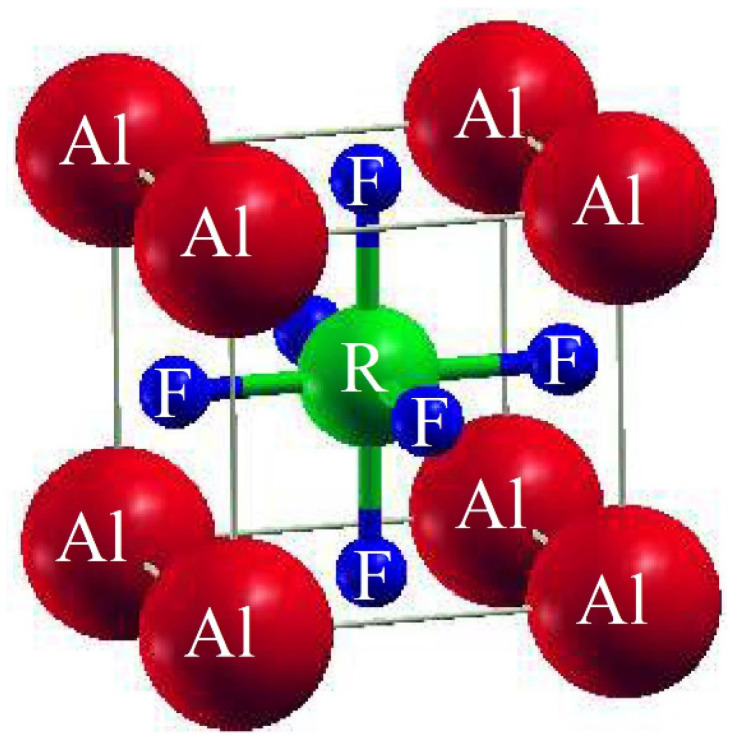
Traditional crystalline structure of compounds AlRF_3_ (R = N, P).

**Figure 2:**
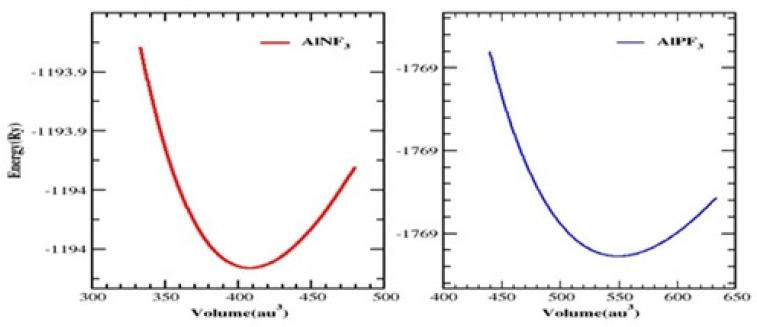
Cubic perovskites AlRF_3_ (R = N, P) and their total energy against volume are plotted.

The unit cell of AlRF_3_ (R = N and P) crystals with space group Pm3m (# 221) had a cubic perovskite structure with a single molecule. The F atoms appeared at (½, ½, ½); the R atoms (R = N, P) at (0, ½, ½), (½, 0, ½) and (½, ½, 0); and the Al atoms at (0, 0, 0), respectively. Figure 1 depicts the arrangement of Al-based fluoro-perovskites cubic crystals. We calculated the total energy of the cell against volume to be around V_0_ (the volume of the building block in stable conditions). Utilizing Birch–Murnaghan’s equation of state [25], the volume optimization technique was used to obtain structural properties. We performed analytical calculations of our acquired locations using Birch–Murnaghan’s equation of state to fit the obtained information and achieve the lowest energy state features, such as an equilibrium lattice length a_o_, a bulk modulus B and its pressure derivative B’. As illustrated in Figure 2, the bottommost energy unit cell could be obtained by reducing the total energy for the appropriate volume of the unit block. The lowest state energy E_o_ was defined as the total minimum energy against volume. The volume was referred to as V_o_, which stands for the optimal least volume. The compounds constructed with the peak optimal energy were expected to be more stable. The ideal structural parameters which we calculated are included in Table 1, including a_o_ (optimized lattice constant), E_o_ (optimized lowest energy state), B (bulk modulus), V_o_ (optimized volume) and B’ (bulk modulus pressure derivative). Since the bulk modulus dropped with an increasing lattice constant, these consequences were consistent with the general trend of this approximation, implying that the estimated conclusions are very precise and genuine.

### 3.2. Electronic Properties

**Figure 3:**
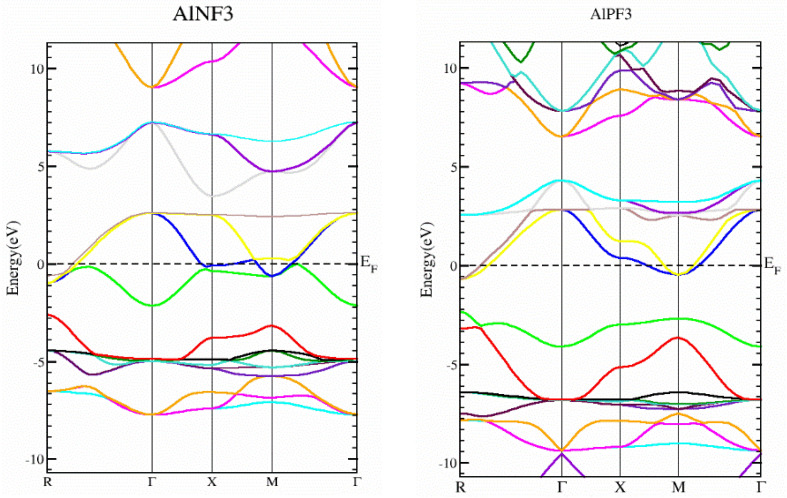
The band structures of AlRF_3_ (R = N, P) compounds, achieved using the Tb-Mbj technique.

**Figure 4:**
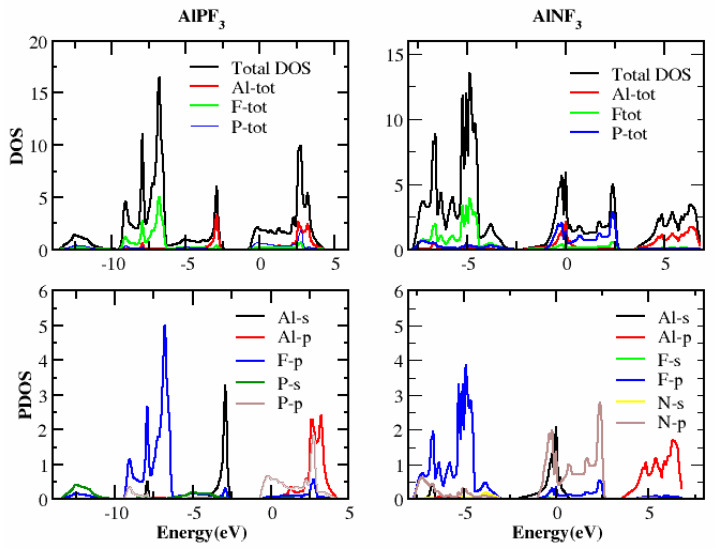
TDOS and PDOS of the compound AlRF_3_ (R = N, P) (Tb-Mbj technique).

In this section, we determined the real diagrams of energy band structures and density of states (DOS) to explore the electronic characteristics of AlRF_3_ (R = N, P) crystals. The basic forbidden region of semiconductors and insulating materials is widely known to be underestimated by local density approximations and GGA simulations [29,30]. The bulk of this is composite because its basic characteristics fail to produce both the exchange-correlation energy and its charge derivatives steadily. To solve band gap underestimation, the TB-mBJ was utilized, as it has been used effectively in recently published works [14,31,32]. Figure 3 illustrates the energy states obtained for AlMF_3_ (M = N and P). The Fermi level energy was selected as the null-energy level at the topmost of the valence band (VB). Since the VB maximum and conduction band (CB) minimum values overlap for AlNF_3_ and AlPF_3_, both are metals. Figure 4 depicts the total density of states (TDOS) and partial density of states (PDOS) for AlRF_3_ (R = N, P) crystals, providing a detailed understanding of the electronic structure. DOS shows how numerous electronic positions contribute to both bands. The perpendicular dashed lines at zero electron volts represent the Fermi energy level E_F_, whereas the DOS ranges from −12.5 to 10 eV. The conduction band is the group of states, which is on the right-side of E_F_ line, while the valance group is to the left. The F-tot, Al-tot, F-p and Al-s states in the VB have prominent contributions.

The energy ranged from −12.0 to −2.5 eV, −4 to −2.5 eV, −12.0 to −2.5 eV and −7.5 to −2.5 eV, for AlPF_3_. In the conduction band, the Al-tot, F-tot, Al-p and P-p states contributed the most in the energy ranges from 2.0 to 4 eV, −1.0 to 4.0 eV, 2.0 to 4.0 eV and −1.0 to 4.0 eV, respectively. In AlNF_3_, the biggest contributions were from F-tot, P-tot, F-p and N-p in the valance band from −8.0 to −2.5 eV, −8.0 to −2.5 eV, −10.0 to 0.0 eV and 10.0 to 0.0 eV, respectively. In the conduction band of the same compound, the highest contributions came from the Al-tot and Al-p states in the energy ranges of 2.5 to 10.0 eV and 2.5 to 8.0 eV, respectively, as illustrated in Figure 4.

### 3.3. Elastic Properties

The elastic property is the reaction to external pressure by a crystal. This property is used to calculate the crystal’s stability. The IRelast package, which was included in Wien2k and is specially built for cubic systems, was used in the calculation of ECs [33]. The three distinct ECs are *C*_11_, *C*_12_ and *C*_44_, which are recycled to achieve ductility and mechanical stability in any particular crystal. Table 2 summarizes these independent constants. For a cubic crystal construction to be mechanically stable, the following ECs criteria should be satisfied: *C*_11_ − *C*_12_ > 0, *C*_11_ > 0, *C*_44_ > 0, *C*_11_ − *C*_44_ > 0, *C*_11_ + 2*C*_12_ > 0 and *B* > 0 [34]. The ECs and *C*_ij_ values determined here reflect the compound’s elastic stability. AlPF_3_ has a *C*_11_ value of 82.03 GPa, which is lower than AlNF_3_’s value of 117.78 GPa. As a result, AlPF_3_ is somewhat harder than AlNF_3_. The tendency of materials to create microscopic cracks is thoroughly connected to the crystal’s elastic anisotropy, represented by A, which may be used specifically in the study of engineering. The value of A was obtained for the aforementioned compounds with the help of the obtained values of *C*_11_, *C*_12_ and *C*_44_, according to Equation (1).

(1) *A =* 2*C*_44_/(*C*_11_ − *C*_12_)

*A* = 1 for an isotropic material, while anything less than 1 implies anisotropy.

Because the values of *A* for both the compounds were not equal to one, we were able to claim that our compounds possessed an anisotropic nature, and that the degree of anisotropy was indicated by the amount of variance. According to Table 2, this was 0.28 for AlPF_3_ and 0.48 for AlNF_3_, which means that AlNF_3_ is more anisotropic in nature. “Shear modulus *G*, Young’s modulus *E* and Poisson ratio *v*” were calculated using ECs, with the help of the following formulas [35,36,37]:

(2) $$E=\frac{9\mathrm{BG}}{G+3B}$$

(3) $$v=\frac{3B-2G}{2\left(G+2B\right)}$$

(4) $$G_{v}=\frac{C_{11}-C_{12}+3C_{44}}{5}$$

(5) $$G_{R}=\frac{5C_{44}\left(C_{11}-C_{12}\right)}{4C_{44}+3C_{11}-C_{12}}$$

(6) $$A=\frac{2C_{44}}{C_{11}-C_{12}}$$

Table 2 shows the *E*, *A*, *v* and *G* values obtained from the equations above. A variety of factors can be recycled to assess the material’s ductility or brittleness. (*C*_11_ − *C*_44_) is the compound’s ductility, while Cauchy’s pressure is the difference between *C*_11_ and *C*_44_ [38]. If *C*_11_ − *C*_44_ has a positive value, the compounds will be ductile, and if it is negative, the compounds will be brittle. Both materials have a positive Cauchy’s pressure value, which is 0.49 GPa for AlPF_3_ and 0.45 Gpa for AlNF_3_, showing that they are ductile. The Pugh ratio, or *B*/*G*, is the second methodology used for determining whether it is brittle or ductile. The greater value of “*B*/*G*” from the critical value of 1.75, the greater the compound’s ductility will be and vice versa [39]. Our compounds were ductile because their values were 44.84 and 9.91 for AlPF_3_ and AlNF_3_, respectively. This means that AlNF_3_ is somewhat less ductile than AlPF_3_. In order to differentiate between the ductile and brittle nature of any compound, T. Frantsevich et al. [40,41], using *v*, obtained 0.26 as the critical value. Therefore, if a material’s “*v*” value is less than 0.26, it will be brittle, and vice versa. Table 2 shows that both ternary AlRF_3_ (R = N, P) compounds had a value greater than 0.26, namely, 0.49 for AlPF_3_ and 0.460 for AlNF_3_, signifying its ductile nature. Finally, we discovered that the AlRF_3_ (R = N, P) compounds are mechanically ductile, anisotropic, tough and fracture-resistant. Based on these results, we can definitely see the elastic properties being used in a range of current electrical devices.

## 4. Optical Properties

We subjected our materials to packets of light with energies ranging from 0 to 14.5 eV, and all the visual properties of our compounds were calculated using the predicted equilibrium lattice constant. The dielectric function ε(ω) was used to determine all optical characteristics.

### 4.1. The Refractive Index

**Figure 5:**
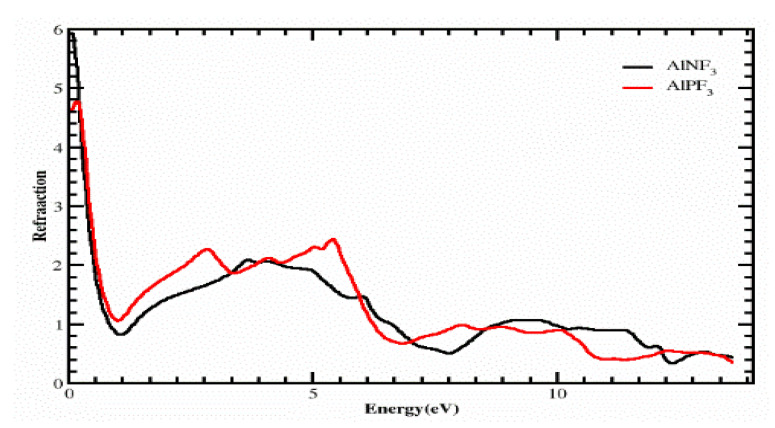
Calculated refractive index of compounds AlRF_3_ (R = N, P).

**Figure 6:**
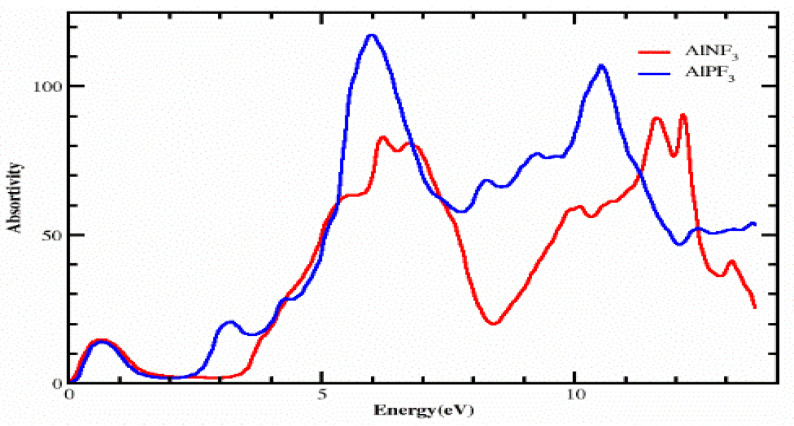
Computed absorption coefficient of complexes AlRF_3_ (R = N, P).

Using ε_1_(ω) and ε_2_(ω), we determined several physical characteristics, such as the refractive index η(ω), optical conductivity σ(ω), absorption coefficient i(ω) and reflectivity r(ω) of our compounds. Figure 5 depicts the computed value of the refractive index 4.3 as 6, and the values of refractive index η(0) = 4.6 at 0 eV for the crystals AlPF_3_ and AlNF_3_, as seen in the refraction spectrum. The curves of Figure 6 clarify that the refractive indexes of the aforementioned compounds slightly deviated from each other. Figure 5 shows that AlPF_3_ had the highest refractive index value of 2.4 and 2.4 at photon energies of 5 and 10 eV, respectively, whereas the peak refractive index of AlNF_3_ was 2.1 at 6.5 eV. We determined the refraction of light from a particular composite based on its refractive index, which was extremely useful for photoelectric purposes. The refractive index was more than 1 (η(ω) > 1) because when a photon enters a compound, it feels hindrance due to the photon–electron interaction, as revealed in the same Figure. The greater the ε(ω) of a substance when it passes through, the more photons are refracted. Each process that raises the electron density of a substance also enhances its refractive index.

### 4.2. The Absorption Coefficient

The absorption coefficient I(ω) was calculated using the dielectric function, as shown in Figure 6. From Figure 6, it is clear that our crystals have appreciable absorption coefficients at energies varying from 0 eV to 14 eV for AlPF_3_ and AlNF_3_. Both of these compounds have essentially identical critical values, which are 0.0 eV for AlPF_3_ and AlNF_3_, respectively. At energy levels of 0.50, 3.30, 4.30, 6.0 and 11.0 eV, AlPF_3_ exhibited absorption peaks of 18.0, 23.0, 30.0, 120.0 and 100.0, respectively. At 0.50, 7.20, 7.0, 10.0, 12.0 and 12.50 eV energy, AlNF_3_ had absorption peaks of 18.0, 85.0, 80.0, 60.0, 85.0, and 86.0, respectively.

### 4.3. The Reflectivity

**Figure 7:**
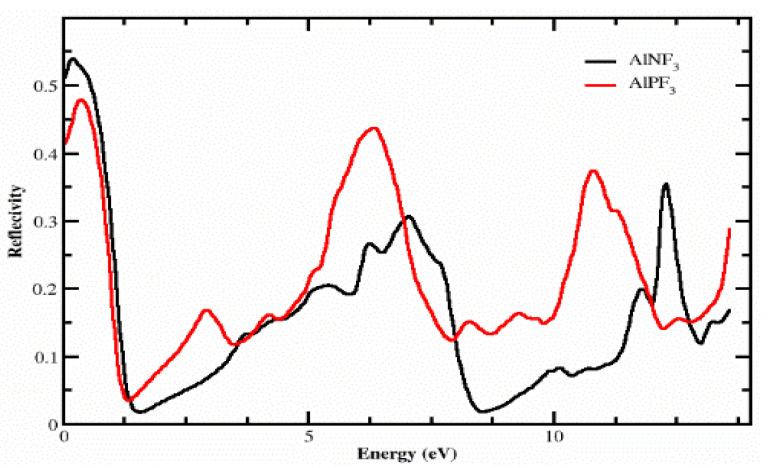
The computed R(ω) of light for the compounds AlRF_3_ (R = N, P).

Figure 7 explains the reflectivity *R*(*ω*), which was estimated from the dielectric permittivity exposed through the variation of energy from 0.0 eV to 14.0 eV. For AlPF_3_ and AlNF_3_, the reflectivity values at zero-frequency *R*(0) were 0.42 and 0.54, correspondingly. As the incident photon energy was enhanced, the reflectivity of both complexes first increased up to approximately 0.4 eV and then declined to 0.09 at 1.0 eV, and after 1.0 eV, it once again rose. Afterward, the values separated, and AlNF_3_ had values of 0.20, 0.35 and 0.4 at 5.2, 6.6 and 10.6 eV, respectively. Similarly, further peaks of 0.19, 0.45 and 0.4 were detected for AlPF_3_ at 2.2, 60 and 10.5–0 eV, respectively. In comparison to AlNF_3_, AlPF_3_ had an extremely low reflectance of 0 eV. After 1.0 eV, it stayed low for AlNF_3_ compared to AlPF_3_ at higher energies, indicating that AlNF_3_ was more transparent than AlPF_3_ in this energy range. The transparency of the material suggested that these crystals may be used to produce lenses.

### 4.4. Optical Conductivity

**Figure 8:**
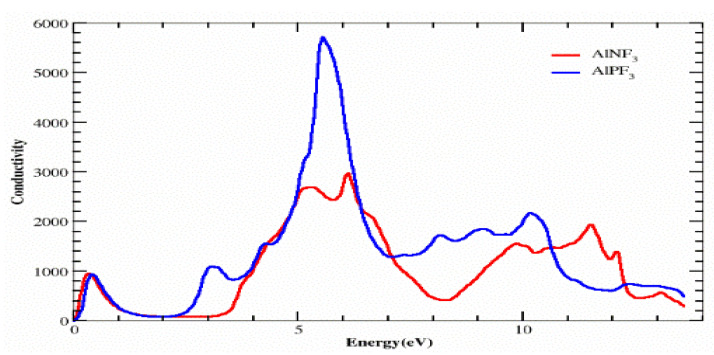
Calculated optical conductivity σ(ω) of AlRF_3_ (R = N, P) compounds.

Photon conduction is mathematically represented as σ(ω), which describes the electrons’ movement in a substance produced by the application of an electromagnetic field. We may study the conductivity σ(ω) using the dielectric function, as revealed in Figure 8.

For both substances, photon conductivity began at 0 eV. Other peaks for AlPF_3_ were 1000, 0, 2200, 5800 and 2800 at 0.4, 2.0, 3.2, 5.9 and 10.3 eV, respectively. Figure 8 depicts the peaks for AlNF_3_ as 1000, 0, 3000, 3100, 1900 and 2000 at 0.4, 2.0, 5.0, 6.3, 9.9 and 11.5 eV, respectively. As a consequence, it was discovered that the AlPF_3_ crystal is optically more conductive at low energy than AlNF_3_.

## 5. Conclusions

In the presented study, we effectively studied the structural, elastic, electronic and optical characteristics of ternary fluoro-perovskite AlRF_3_ (R = N, P) crystals. The most accurate and recent results are as follows. Based on improved structural parameters, we determined that AlRF_3_ (R = N, P) is cubic and structurally stable. The IRelast program was applied to guess flexible constraints, such as fundamental ECs, “anisotropy factor, Poison ratio, ductility, Cauchy’s pressure, shear modulus, Pugh ratio and Young modulus”. Based on these basic elastic properties, it was determined that the aforementioned crystals were elastically stable, anisotropic, scratch-resistant and ductile. As a result of these discoveries, we are confident in the use of these materials in a wide range of current electrical technologies. To investigate the basic electrical properties of our crystals, the TB-mBJ approximation was adopted. The chemicals AlPF_3_ and AlNF_3_ were revealed to be conductors. F-tot, Al-tot, F-p and Al-s states in the VB contributed the most to the DOS, with energies ranging from −12.0 to −2.50 eV, −4 to −2.50 eV, −12.0 to −2.50 eV and −7.5 to −2.5 eV for AlPF_3_. For AlPF_3_, the biggest contribution in the conduction band came from the Al-tot, F-tot, Al-p and P-p states, with energies ranging from 2.0 to 4.0 eV, −1.0 to 4.0 eV, 2.0 to 4.0 eV and −1.0 to 4.0 eV, respectively. In contrast, the greatest contributions to AlNF_3_ were from F-tot, P-tot, F-p and N-p, from −8.0 to −2.50 eV, −8.0 to −2.50 eV, −10.0 to 0 eV and 10.0 to 0 eV, respectively. In the same compound’s conduction band, the greatest contributions came from the Al-tot and Al-p states, with energies ranging from 2.50 eV to 10.0 eV and 2.5 to 8.0 eV, respectively.

## Figures and Tables

**Table 1:** Optimized structural parameters of AlRF_3_ (R = N, P) obtained using Birch–Murnaghan’s energy versus volume equation.

Compounds	a_o_ (Å)	B (GPa)	B’	V_0_ (a.u^3^)	E_0_ (Ry)
**AlNF_3_**	3.92	81.27	4.89	407.45	−1193.97
**AlPF_3_**	4.34	64.45	4.83	549.82	−1769.05

**Table 2:** Computed values of ECs, *A*, *G*, *v* and *B*/*G* for the AlRF_3_ compounds (R = N, P).

Compounds	AlPF_3_	AlNF_3_
*C*_11_	82.03	117.78
*C*_12_	65.94	64.20
*C*_44_	2.22	12.91
*G*	3.83	17.37
*A*	0.28	0.48
*v*	0.49	0.46
*B*/*G*	44.84	9.91

## Data Availability

Not applicable.
